# Recent Progress in Fluorescent Probes For Metal Ion Detection

**DOI:** 10.3389/fchem.2022.875241

**Published:** 2022-04-13

**Authors:** Luanjing Li, Jiahe Wang, Shihan Xu, Chunxia Li, Biao Dong

**Affiliations:** ^1^ Sdu-Anu Joint Science College, Shandong University, Weihai, China; ^2^ State Key Laboratory on Integrated Optoelectronics, College of Electronic Science and Engineering, Jilin University, Changchun, China; ^3^ Department of Bioengineering, University of Washington, Seattle, WA, United States; ^4^ Institute of Frontier and Interdisciplinary Science, Shandong University, Qingdao, China

**Keywords:** fluorescent probes, metal ions, cell imaging, sensors, quantum dots

## Abstract

All forms of life have absolute request for metal elements, because metal elements are instrumental in various fundamental processes. Fluorescent probes have been widely used due to their ease of operation, good selectivity, high spatial and temporal resolution, and high sensitivity. In this paper, the research progress of various metal ion (Fe^3+^,Fe^2+^,Cu^2+^,Zn^2+^,Hg^2+^,Pb^2+^,Cd^2+^) fluorescent probes in recent years has been reviewed, and the fluorescence probes prepared with different structures and materials in different environments are introduced. It is of great significance to improve the sensing performance on metal ions. This research has a wide prospect in the application fields of fluorescence sensing, quantitative analysis, biomedicine and so on. This paper discusses about the development and applications of metal fluorescent probes in future.

## 1 Introduction

Metal cations and anions play an important role in the versatile physiological and pathological processes, including metabolism, osmotic regulation, catalysis and so on. It is well known that normal biological events can be adversely affected by maladjustment on the levels of certain ions in organisms (Park et al., 2020). It is because of these ions has certain pathophysiological significance, so we explore how these ions detection in biological systems of sensitive and selective is important.

In order to detect and quantify ions, researchers are committed to developing appropriate chemical sensors. Fluorescence in combination with appropriate probes is a good way to measure metal ions, because fluorescence has certain advantages, such as being faster, less destructive and more sensitive, which can present information about the location and quantity of the target (Li et al., 2017). Fluorescence probe method means that the photophysical properties of probe molecules change obviously before and after the specific binding between probe and analyte, so as to detect the change of fluorescence signal, realizing the detection of different molecular or ion content in the organism or the environment (Xu et al., 2016). In general, for the fluorometric determination of cations or anions, the sensor must consist of two components: a fluorescent carrier and an ionic carrier, which may be independent species or covalently linked on a molecule.

Fluorescent probes can be combined with bioluminescence imaging technology to achieve *in vivo* detection at the cellular level or animal level, which is considered to be the most potential tool for studying different components in the organisms. Fluorescent probe for metal ions, therefore, carries on the synthesis and design gradually become the research hot spot. It is of great significance in chemistry, biology, clinical medicine and agriculture to search for novel organic molecular recognition carriers with high selectivity and to design a novel fluorescent probe for metal ion detection (Xu and Xu, 2016). To have an extensive overview of present studies, we summarized popular fluorescent probes from literatures, classified them according to the types of ions being detected, and presented their structures and strategies. In addition, we focus on numerous strategies to improve the selectivity of fluorescent probes, including metal-organic backbones, central hydrophilic external hydrophobic strategies, etc., which will provide additional insights for biomedicine.

**FIGURE 1:**
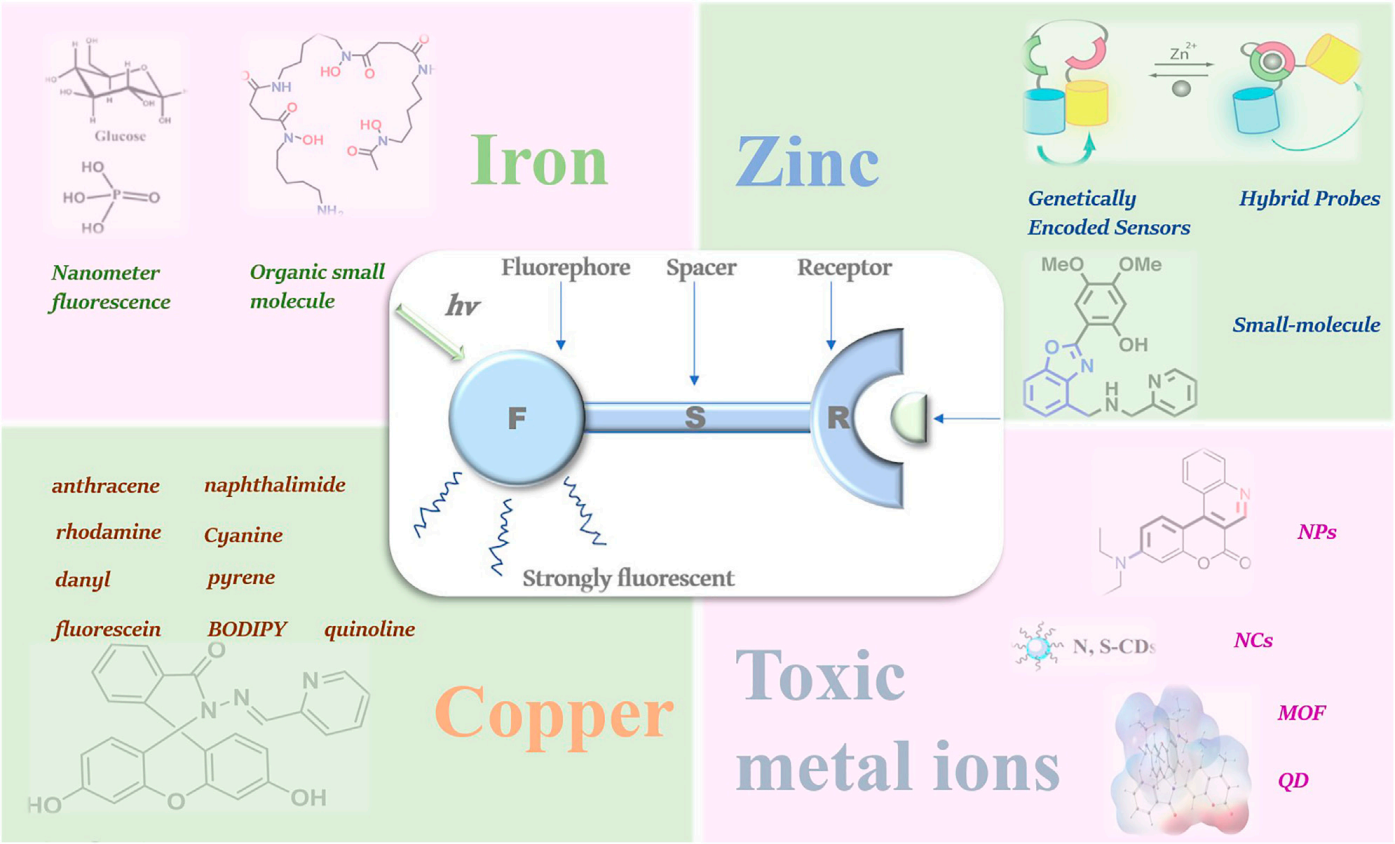
Various types of fluorescent probes for metal ion detection.

In this review, the latest research progress of fluorescent probes for iron, copper, zinc, mercury, lead, chromium and other metal ions are summarized (Figure 1), and the development trend and application prospect of this field are also discussed. Basic information on the fluorescent probes for the detection of various ions is listed in Table 1.

**TABLE 1:** List of methods of fluorescent probe detection of metal ions.

Target	Probe	LOD/μM	Real Sample	Ref.
Fe^3+^	N, P-CDs	1.8 × 10^–3^	Human Serum, Living Cells	8
	N-CDs	1.14	Vero cells	9
	Phe-CDs	0.72	water samples	11
	KN-CDs	0.85	—	12
	N-GQDs	9 × 10^–2^	aqueous media	13
	TPC	0.2 × 10^3^	water samples	24
	LaOBr/DAT	0.3	living cells	25
Fe^2+^	CDs	—	living cells	27
	MOFs	—	aqueous environments	28
	ACQ	—	living cells	30
	FeP1	1.8 × 10^–2^	living cells, physiological saline	33
	P FeaD	0.46	Live cells, cosmetics	34
Cu^2+^	turn-on red-emitting	4 × 10^–3^	living plant tissues, living zebrafish	40
	DCM-Cu	2.54 × 10^–2^	living MCF-7 cells	41
	CdTe/ QDs	20	real samples	42
	CQDs	5	living cells	43
	BTPPA	0.506	real samples (tea, fish, crab meat)	49
Zn^2+^	UCNPs	—	real biological samples	61
	NR-Zn	0.131	Hela cells	69
Hg^2+^	N,S-CDs	0.083	living cells	83
Pb^2+^	MNPs	5.7 × 10^3^	tea and waste water	90
Cd^2+^	6-(dimethylamino)	0.515	in living cells	112

## 2 Probes For Iron

### 2.1 Probes For Detecting Fe^3+^

Fe^3+^ is considered among the most important metal ions in biological systems and exerts an unparalleled role in many biological processes one of the most vital metal ions in biological systems, such as RNA and DNA synthesis, metabolism and so on. To develop new fluorescent probes having low levels of cellular toxicity, good biocompatibility and high solubility in water is becoming more and more important and urgent.

Lytton and co-workers (Lytton et al., 1992) put forward the earliest reported about an iron fluorescent sensor, which is on the basis of the iron carrier desferrioxamine B (DFO) model, connect to the fluorescent carrier 7-nitrobenz-2-oxa-1, 3-diazole (NBD). By detecting and monitoring Fe^3+^ performance in solution, the NBD-DFO model proves its advantage in monitoring iron under various conditions of iron imbalance diseases. For the past few years, various fluorescent probes developed for selective detection of Fe^3+^ can be divided into four categories according to the fluorescence signal processing: Turn-Off, Turn-on, ratiometric and chemodosimeters.

#### 2.1.1 Nanometer Fluorescence Probes

For the exploration of the iron ion, nano fluorescent probe plays an important role. Among them, one of the most well-studied materials is semiconductor nanoparticles, also known as quantum dots. In recent years, carbon dots (CDs) are widely used in fluorescent biological imaging and sensing (Zhu et al., 2013). However, the low quantum yield (QY) of CDs severely restricts its development and application. Chemically doped heteroatoms can effectively adjust electron density to improve the QY value of CDs. Therefore, the photochemical and physicochemical properties of carbon dots (CDs) can be efficiently regulated by chemical doping heteroatoms (Guo et al., 2015).

**FIGURE 2:**
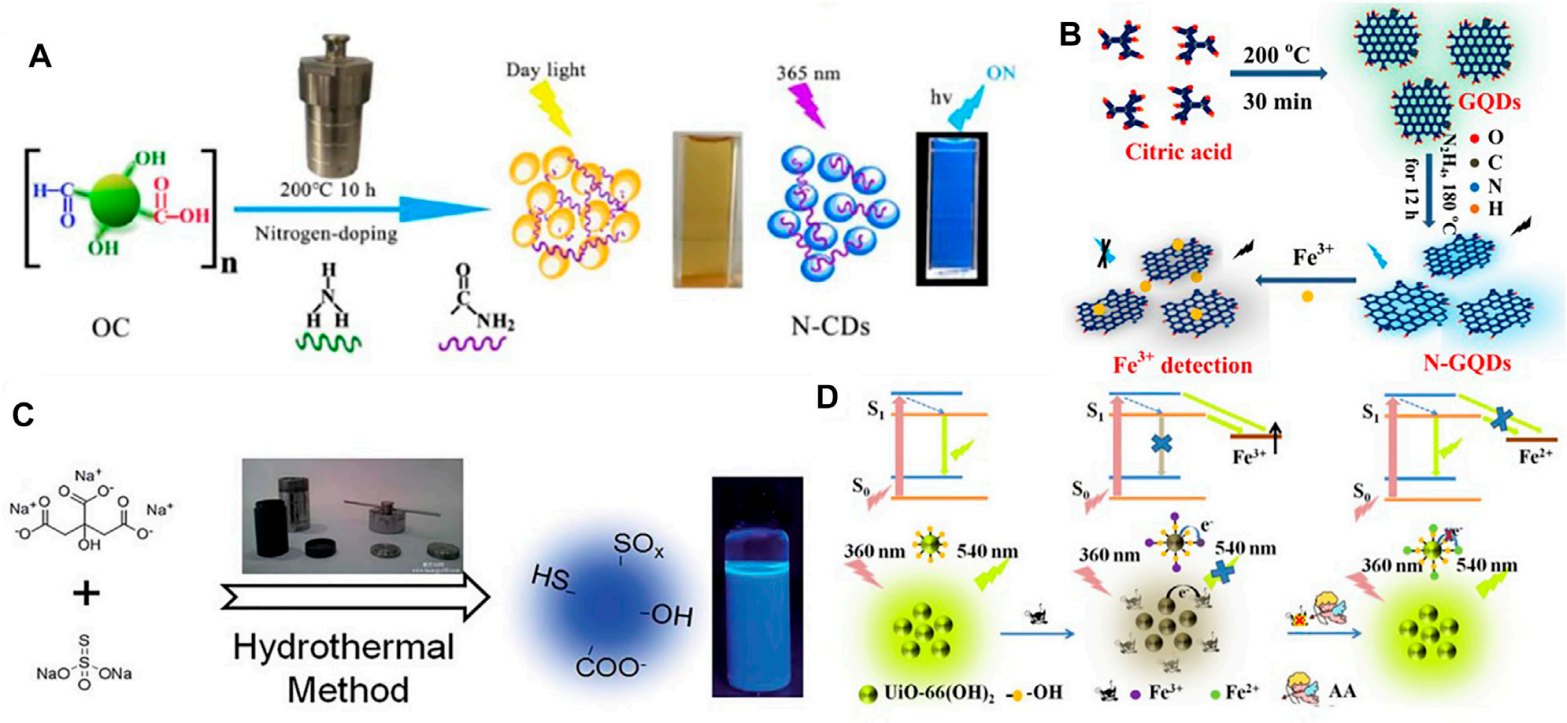
**(A)** The schematic diagram of preparation of fluorescent N-CDs *via* hydrothermal treatment (Liu Z. et al., 2020); **(B)** Schematic representation of the procedure for synthesizing N-GQDs and the detection of Fe^3+^ (Ju and Chen, 2014); **(C)** Scheme of the synthesis of S-doped C-dots with blue luminescence (Xu Q. et al., 2015); **(D)** Schematic illustration the “on-off-on” fluorescent detection of Fe^3+^, AA using UiO-66-(OH)_2_ as sensor (Wang H. et al., 2021).

Shi *et al.* (Shi et al., 2016) synthesized N, P-CDs multi heteroatom (nitrogen and phosphorus co-doped carbon nanodots) fluorescence sensor, which has low cytotoxicity and high photostability, and measured its sensitivity to Fe^3+^. Liu *et al.* (Liu Z. et al., 2020) synthesized the non-toxic nitrogen-doped fluorescent carbon dots (N-CDs) that can detect ferric ions, at the same time can be used for fluorescent probe of Vero cell biological imaging (Figure 2A). Du *et al.* (Du et al., 2021) demonstrated that off-on switch fluorescent probe based on yellow emission carbon dots (y-CDs) provides a highly sensitive and selective method for the detection of ferric ions. Pu *et al.* (Pu et al., 2020) proposed a static quenching mechanism about the phenylalanine carbon dots (Phe-CDs), which kept its outstanding fluorescence intensity despite its extreme pH values. Atchudan *et al.* (Atchudan et al., 2021) reported that they synthesized fluorescent carbon dots (KN-CDs) by hydrothermal carbonization method as a good fluorescence sensor of Fe^3+^ based on the closing sensor of Fe (III) ions. Ju *et al.* (Ju and Chen, 2014) synthesized environmentally friendly nitrogen-doped graphene quantum dots (N-GQDs) for the unlabeled Fe^3+^ ions detection in various actual water (Figure 2B). Qi *et al.* (Qi et al., 2019) designed and demonstrated the promise of nitrogen-doped carbon quantum dots (N-CQDs) as probes for iron ions. Zhang *et al.* (Zhang Y. et al., 2021) obtained B_1_N_2_CQDs (core-shell carbon quantum dots), which has been proved that its detection of Fe^3+^ can be applied to both endocellular scenarios in biological system and river water samples due to its high stability and fluorescence quantum yield. Xu *et al.* (Xu Q. et al., 2015) synthesized Sulfur-doped carbon dots (S-doped C-dots), which can also be used to detect iron ions efficiently (Figure 2C).

Qu *et al.* (Qu et al., 2013) took advantage of dopamine for light source synthetic photoluminescence carbon nanoparticles (CNPs), which can be used as a very powerful fluorescence sensitive stage and has been successfully applied to iron ion detection in some water samples. Based on hydroxy functional metal organic skeleton (MOF) UiO-66-(OH)_2_, Wang *et al.* (Wang H. et al., 2021) also proposed a “on—off—on” fluorescent switch nano prober (Figure 2D). Dong *et al.* (Dong et al., 2021) synthesized the NIR PL of GSH-capped gold nanoclusters (GSH-AuNCs), which exhibits excellent sensing performance.

#### 2.1.2 Organic Small Molecules

Xu and co-workers (Xu H. et al., 2015) obtained a owns two one-dimensional channel unparalleled of three-dimensional TB - BTB (benzene-1, 3, 5-tribenzoate) framework for high sensitivity detection of Fe^3+^. Using an EuL3 (L = 4'-(4-carboxyphenyl)-2, 2':6', 2 "-tripyridine) fluorescence sensor, Zheng and co-workers (Zheng et al., 2013) designed a portable daily life iron ion test paper. Azmi *et al.* (Al-Azmi and John, 2021) produced a tricyanofuran hydrazone (TCFH) optical probe for better recognition of Fe (III) ions in the hydro environment. Zhang (Zhang et al., 2018) reported a new fluorescence and specific color probe containing both deoxycholic acid and rhodamine molecules and used the spectrum to achieve the measurement of iron ions.

**FIGURE 3:**
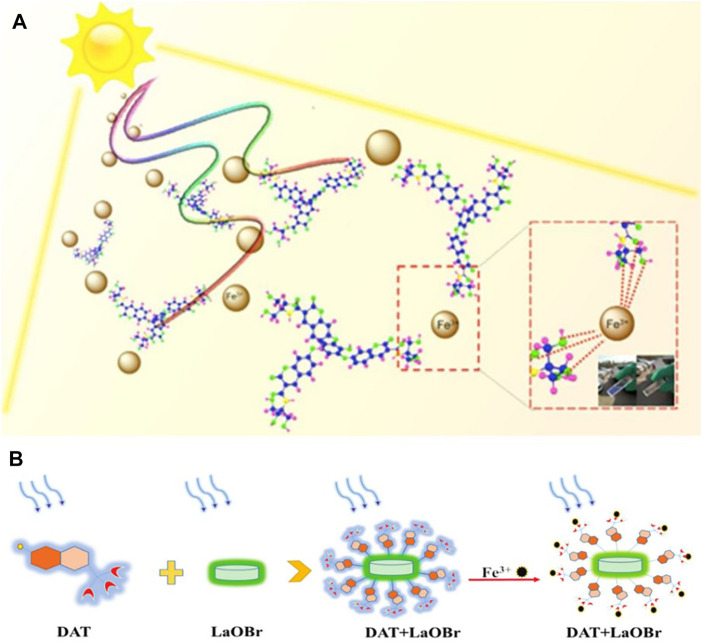
**(A)** The trimeric phenolic coumarin (TPC) designed through the internal hydrophobic-external hydrophilic strategy achieves greatly enhanced Fe^3+^ sensing performance, and can be used for naked eye detection with sunlight excitation (Liu J. et al., 2020); **(B)** A schematic diagram showing the preparation of the LaOBr/DAT [N-(2-hydroxy-1,1-bis (hydroxymethyl) ethyl)-7-hydroxycoumarin-3-carboxamide] composite and the mechanism of Fe^3+^sensing (Sun et al., 2020).

In order to achieve better sensing performance and water-solubility of organic molecular probes, Liu *et al.* (Liu J. et al., 2020) proposed a method with great potential and development space. They linked the inner hydrophobic molecule tribromophenol with the outer hydrophobic molecule coumarin on the basis of the original iron fluorescence probe (Figure 3A). Besides, in view of the water-soluble dilemma of organic small molecules, Sun *et al.* (Sun et al., 2020) also adopted a similar structural strategy, and developed a unique vanilloid fluorescent probe for the assay of iron ions by increasing the nucleophilic substitution reaction of LaOBr (Figure 3B). Designing composite materials is of great significance to the change of the water solubility of organic small molecules, and it can also improve the probe’s sensitivity to ions.

High levels of trivalent iron ions can cause many diseases, including cancer, organ disorders of the heart, liver and pancreas, hepatitis, Parkinson’s disease, Alzheimer’s disease, etc. Therefore, the development of fluorescent probes for iron ions has greatly facilitated biomedical imaging in human cells and has contributed to disease diagnosis.

In last several years, lots of researchers have designed different methods of novel iron (III) fluorescence sensors, and successfully synthesized sensors for monitoring the concentration of environmental iron and studying iron migration in various microbial species. Most sensors are derived from the principle of fluorescence quenching, so future probes should be preferentially designed for absorption at longer wavelength to avoid the internal filtering effect of iron (III) absorption in the UV-vis region.

### 2.2 Probes For Detecting Fe^2+^

Although the iron in the cells has two forms of Fe^2+^ and Fe^3+^, it mainly exists with the form of Fe^2+^ because of the reductive microenvironment of cells (Ma et al., 2021). On the one hand, it is difficult to establish a highly specific probe for the detection of Fe^2+^, because the different oxidation states of Fe^2+^ and Fe^3+^ vary with each other. On the other hand, due to the strong force between paramagnetic Fe^2+^ and fluorophore, fluorescence quenching is usually induced, resulting in part of the constructed probes being “on-off” type, which is not easy to observe due to the influence of spontaneous fluorescence of organisms (Wei et al., 2020). Currently, the Fe^2+^ fluorescent probes mainly include N-oxides, nitroxyl radicals, endoperoxides, bionic ligands, heavy metals, imines and so on.

**FIGURE 4:**
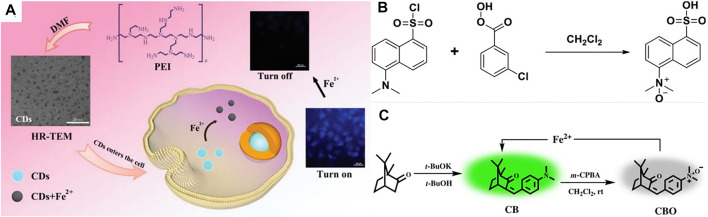
**(A)** One-pot hydrothermal synthesis of fluorescent CDs as a highly efficient “on-off” fluorescent probe for the rapid detection of intracellular Fe^2+^ (Zhang X. et al., 2020); **(B)** The synthesis route of probe FeP1 (Khatun et al., 2020); **(C)** The synthesis route of probe CBO (Liang et al., 2021).

In order to achieve high-efficiency sensing of Fe^2+^, Yan *et al.* (Yan, 2017) proposed a dual-correspondence luminescence probe with optimized metal-Organic Framework (MOF) as the main body. The special feature of this probe is the use of various luminescent substances, such as lanthanide ions, carbon quantum dots, etc. Xu *et al.* (Xu and Yan, 2015) also synthesized a layer similar to MOF [MIL-124, or Ga_2_ (OH)_4_ (C_9_O_6_H_4_)], and further designed and developed a probe to detect Fe^2+^ ions. For Fe^2+^ detection, Yang *et al.* (Yang et al., 2021) reported on the aggregation-induced emission (AIE) based probe named QM-Fe, and verified the feasibility of Fe^2+^ detection in viable cells. Zhang *et al.* (Zhang D. et al., 2021) gained a new spiropyran-based fluorescent probe to detect Fe^2+^ ion. The results show that the addition of Fe^2+^ with the probe solution improved the magnetic intensity of fluorescence by 6-fold. Wei *et al.* (Wei et al., 2020) developed a “on-off” fluorescent probe based on a carbon point (CDs), which has been efficiently used to detect Fe^2+^ in BSA solution, tap water and living cells (Figure 4A). In order to realize real-time detection of unstable ferric divalent ions in living systems, Long *et al.* (Long et al., 2018) designed a fluorescent probe of coumarin by using a unique cyclization reaction. Gao *et al.* (Gao et al., 2020) constructed a practical and novel Fe^2+^ fluorescent probe *via* a unique strategy with Fe^2+^-induced reducing reaction, which can act in different monitoring of the environment, such as living cells. Khatun *et al.* (Khatun et al., 2020) developed a kind of “Turn-On” probe P-Fe (II), which is used to measure the exact amount of ferrous ion (Fe^2+^) in cosmetics or living cells (Figure 4B). Liang *et al.* (Liang et al., 2021) developed a new camphor-based fluorimetic probe (CBO) with a natural monoterpene ketone camphor as a base material for the detection of Fe^2+^ and it can be easily observed by fluorescence of Fe^2+^ ions in some animals and in plant cells Fe^2+^ imaging (Figure 4C).

By stimulating the two-photon microscope fluorescence imaging at 680 nm, Yang *et al.* (Yang et al., 2019) reported for the first time a novel intramolecular charge transfer (ICT) based two-photon, near-infrared (NIR) -enabled fluorescent probe for detecting Fe^2+^, and applied to the actual living cells. In view of the test organisms or the ferrous ions in aqueous environment, Zhang *et al.* (Zhang X. et al., 2020) on the basis of nitrogen oxides reduction reaction, invented and created a new kind of “off—on” fluorescent probes the NT—Fe (4-Amino-1, 8-naphthalimide), which greatly improved the efficiency of Fe^2+^ detection in zebrafish.

During recent years, although there are not numerous reports on the detection of Fe^2+^ by the methods of fluorescent probes, some progress has been made. It can be roughly divided into “off-on” type Fe^2+^ fluorescent probes, “on-off” type Fe^2+^ fluorescent probes, ratio type and other types of Fe^2+^ fluorescent probes. These in-depth exploration and research will be of great significance for in-depth understanding of the specific functions of Fe^2+^ in organisms and the mechanism of action on diseases.

## 3 Probes For Detecting Copper

As we all know, copper ion (Cu^2+^) is another heavy metal ion found in very high concentrations in our people’s body. As a cofactor of many enzymes, copper ion is inseparable from various enzymatic catalysis and electron transfer processes, and it occupies a unique and key position in various physiological processes. Therefore, if copper homeostasis is out of whack, it can lead to a lot of neurodegenerative diseases that we don’t expect. In order to achieve an optimal measurement performance for copper ions, small polymer fluorescent probes have been extensively used in the procedure of microscopic image analysis due to their certain uniqueness.

So far, methods about the Cu^2+^ ions’ detection using small molecular fluorescent probes have generally included the UV-toO-NIR regions of anthracene, danyl,pyrene, quinazoline, cyanine dyes, naphthalimide, quinoline, rhodamine, fluorescein, BODIPY and so on (Sivaraman et al., 2018). However, many significant properties of fluorescent probes used in living cells and *in vivo* still need to be improved, such as slow response speed, high detection limit and poor selectivity (Kar et al., 2013).

**FIGURE 5:**
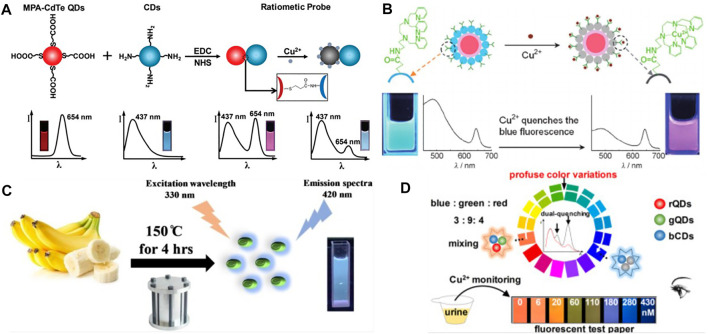
**(A)** Schematic illustration of the formation of the dual-emission ratiometric fluorescence probe and the visual detection principle for copper ions (Wang et al., 2016); **(B)** Dual-emission fluorescent sensing of Cu^2+^ ions based on a CdSe@C-TPEA nanohybrid (Zhu et al., 2012); **(C)** Schematic representation of NS-CQDs synthesis synthesizing NS-CQDs from banana juice (Chaudhary et al., 2020); **(D)** Schematic illustration of the visual detection principle for Cu^2+^ using the tricolor probe (Cai et al., 2018).

Recently, Zhou *et al.* (Zhou et al., 2021) designed an open red fluorescent probe that can be used well and efficiently to detect copper ions (Cu^2+^) in some food samples and live zebrafish. Besides, Copper (Cu) has also been found to be indispensable and extremely important in the process of oxygen-containing photosynthesis in biological systems. Li *et al.* (Li et al., 2021) developed a novel “turn on” NIR probe DCM-Cu whose basic is DCM for the detection of copper ion (II). Due to its wonderful sensitivity and low cytotoxicity toward Cu^2+^ with a Stokes shift of 140 nm, the probe has been widely used by scholars. What’s more, by covalently linking carboxylate-modified red fluorescent cadmium telluride (CdTe) quantum dots (QDs) with fluorescent blue carbon nanodots functionalized with amino groups (CDs), Wang *et al.* (Wang et al., 2016) developed a ratio fluorescence nanosensor with good performance for Cu^2+^ detection (Figure 5A). In addition, Zhu *et al.* (Zhu et al., 2012)also adopted a ratio fluorescence detection method for copper ions, in particular, the CdSe@C quantum dots (QDs) with different fluorophore dual-emission were selected. This probe ion recognition higher and better stability (Figure 5B). Lin *et al.* (Lin et al., 2014) successfully designed a new type of high-fluorescence metal-organic structure (MOFs) for the determination of copper ion content in water in the environment. This strategy utilized a branched chain poly-ethylamine encapsulated carbon quantum dot material, thus improving the fluorescence quantum yield and detection effect. By using mesoporous silica (MS) spheres as the material of nanometer reactors, Zong *et al.* (Zong et al., 2014) improved the fluorescence probe of carbon point (CDs), and made a contribution to the detection of copper ions by synthesizing CDs as fluorescence probe by “off-on” method. Wang *et al.* (Wang L. et al., 2020) implemented the chemical probe formation of the copper (II) complex L- Cu^2+^ as a dual-channel recognition probe, allowing one to identify dramatic changes in color with the naked eye. Yao *et al.* (Yao et al., 2013) designed a ratiometric fluorescent probe based on hybridized dual-emission quantum dots (QDs) and demonstrated that it can visualize real-time monitoring of copper ions in natural environmental water sources, greatly improving efficiency. Ye *et al.* (Ye et al., 2015) first reported the use of a cadmium pamoate metal-organic framework as a bifunctional fluorescent sensor for the detection of trace amounts of 2, 4, 6-Trinitrophenol (TNP) and Cu^2+^, a study that greatly improved the sensitivity of the fluorescent probe for the recognition of copper ions. For the detection of copper ions in actual samples, Tang *e*t al. (Tang et al., 2020) developed a pyridine amide (BTPPA) with aggregation-induced emission (AIE) properties as a probe, which achieved by aggregating switching strategies, through “on-off-on” variation of emissions.

Fluorescent probes for the detection of Cu(II) in water samples are also accessible from natural sources, such as Chaudhary *et al.* (Chaudhary et al., 2020)’s synthesized and published highly fluorescent N,S co-doped CQD (NS-CQD) at an excitation wavelength of 330 nm with an enhanced quantum yield (32%) (Figure 5C). For monitoring and identifying Cu^2+^, Wang et al. (Wang Z.-G. et al., 2020) developed a novel colorimetric/fluorescent probe (7-(diethylamino)-2-oxo-2H-chromen-3-yl)methylene)-4-(dimethylamino) benzohydrazide (HL). Furthermore, in order to improve the continuity of the process when detecting copper ions, Mohammadi *et al.* (Mohammadi and Ghasemi, 2020) developed a novel pyrimidine-based chemosensor (PyrCS) and was able to achieve an intuitive vivid colorimetric response for observation in a specific pH range. Song *et al.* (Song et al., 2018) designed a dual emission ratio fluorescence sensing membrane for copper ion detection. This double emission film managed to fabricate the chitosan, graphite carbonitride (G-C_3_N_4_) and Gold nanoclusters (Au NCs). The film has high sensitivity and portability, which opens up a new way for the detection of copper ions in the environment. Ranee *et al.* (Ranee et al., 2018) synthesized quinoline based novel fluorescent probes for the selectivity and sensitivity of detecting Cu^2+^ ions. Kumar *et al.* (Gujuluva Gangatharan et al., 2018) synthesized an easily available and portable “off-off” colorimetric and fluorescent probe with excellent results in the application of trace Cu^2+^ ions in real water samples. Cai *et al.* (Cai et al., 2018) reported a fluorescent test paper probe consisting of three different emitting quantum dots including blue (bCDs), green (gQDs), and red (rQDs), which enables a simple and rapid detection of Cu^2+^ ions in human urine by observing the color of the filter paper (Figure 5D). Abnormal levels of copper ions in living organisms can also cause many neurological related diseases, and therefore it is of great importance for biomedicine to have a variety of copper ion fluorescent probes for use in living cells.

## 4 Probes For Detecting Zinc

Zinc is the next most prevalent of the transition metal ions in the human body. In small amounts, Zn^2+^ is beneficial to people’s health, but at higher concentrations, it appears toxic. Zinc imbalances have been linked to serious neurological diseases, such as Alzheimer’s and Parkinson’s (Xu et al., 2012). So far, people have developed a variety of Zn^2+^ fluorescent sensors, and successfully applied in living cells, hippocampal slices, and Zn^2+^ imaging in zebrafish, especially Lippard and Nagano. In more recent years, zinc ion fluorometric probes have been arranged under different columns of fluorescent architecture, including unquinoline, rhodamine, naphthalene, coumarin, naphthalimide, pyrene, luciferin, derivatives of phenol and several other fluorophore groups (Wang F. et al., 2021).

Walkup *et al.* (Walkup et al., 2000) first prepared a novel, highly affinity, selective, membrane permeable Zn^2+^ fluorescence sensor. Roy *et al.* (Roy et al., 2007) investigated a DFP based sensor for the first time, which can be used as a fluorescent probe for the detection of zinc ions and applied under certain physiological conditions. Peng *et al.* (Peng et al., 2015) designed and synthesized a chromophore-based up-conversion nanoparticle (UCNPs) nano system as a fluorescent probe for Zn^2+^ by combining chromophore groups and lanthanide-doped UCNPs together. This method was demonstrated to significantly improve the efficiency of divalent Zn ion detection in specific animals, such as zebrafish. Kim *et al.* (Kim et al., 2013) reported a unique fluorogenic Zn^2+^ chemosensor based on a cap-typed tripodal Schiffbase. Zhou *et al.* (Zhou et al., 2010) designed a hydrazone-pyrene-based fluorescent probe with simplicity and efficiency in order to improve the selectivity of the fluorescent probe for Zn ions. Then they further expanded the application scenario of the probe and verified the feasibility of detecting Zn^2+^ ions in pancreatic β-cells, which made a contribution to the biomedical field. Hagimori *et al.* (Hagimori, 2013) reported that novel fluorescent probes based on pyridine-pyridone possess low molecular weight for zinc ion detection. Price *et al.* (Price et al., 2018) reported AQA-F, a fluorescent probe that can be used to detect zinc ions in prostate and prostate cancer cell lines *in vitro*. Zhang (Zhang et al., 2012) developed a pyrazoline based fluorescent probe and verified that this probe showed 40-fold enhanced fluorescence for Zn^2+^ compared to other metal ions, i.e., good selectivity for Zn ions, and that it could be well applied in living neuronal cells.

**FIGURE 6:**
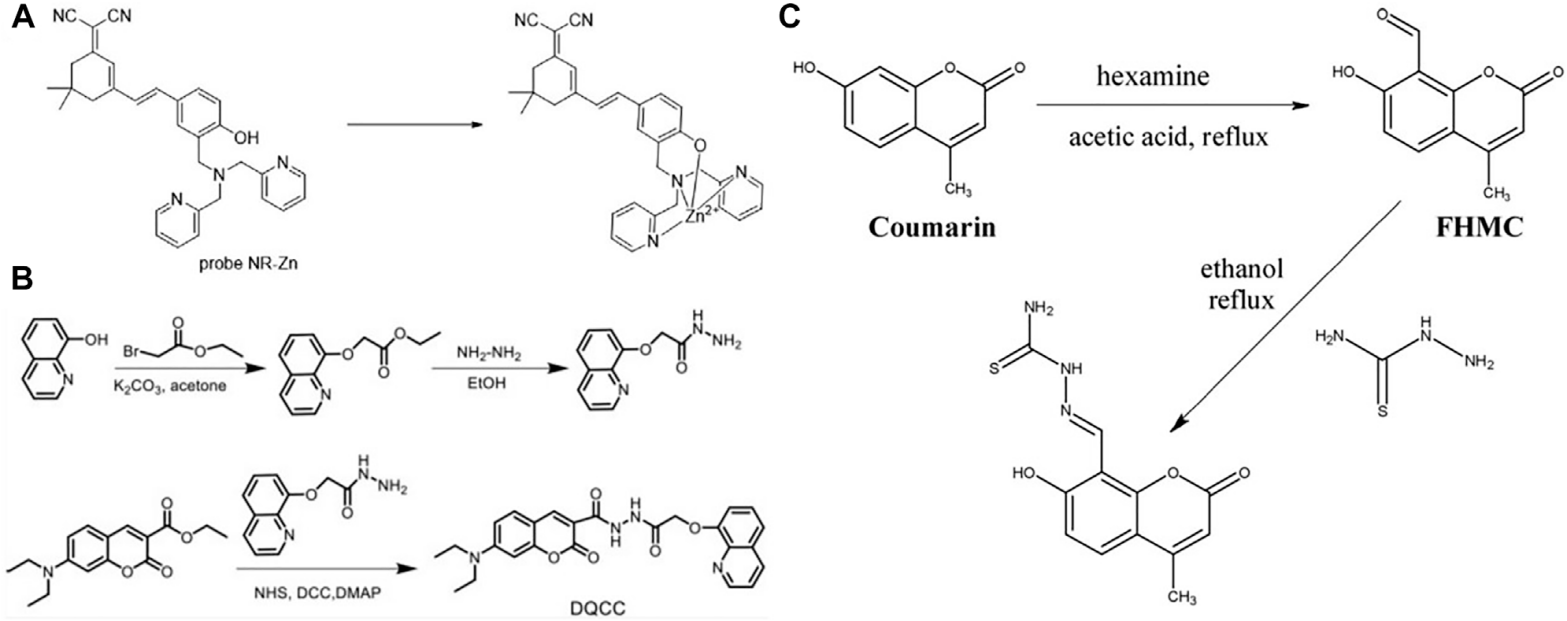
**(A)** The structure of the probe NR-Zn and its interaction mode with Zn (II) ion (Zhang Y. et al., 2020); **(B)** The synthesis route of DQCC (Kang et al., 2019); **(C)** The synthesis route of probe 1 (Wang et al., 2018).

Zhang *et al.* (Zhang et al., 2016) reported two near-infrared fluorescent probes based on the fluorophore platform corresponding to Rhodol functionalized by dimethylamine Zn (II) binding groups, and this probe enables the detection of Zn (II) ions produced by intracellular metalloproteins. In addition to this, Fang *et al.* (Fang et al., 2016) similarly synthesized and studied two NIR fluorescent probes (A and B), but chose different material bases. One is a semicyano structure attached to dimethylamine (DPA) and the other is a dimethylamine derivative with pyridine substituted by pyrazine, which are very effective in detecting zinc ions in living cells. Among the various near-infrared fluorescence (NIR) probes, Zhang *et al.* (Zhang Y. et al., 2020) synthesized a near-infrared fluorescence (NIR) probe NR-Zn consisting of a dicyanoisophorone derivative and a dimethylamine molecule in a structure (Figure 6A). So far, this probe has proved to be successfully applicable for zinc ion recognition in Hela cells. Kang *et al.* (Kang et al., 2019) designed a sophisticated fluorescent probe test paper in the acyl hydrazine linkage mode that allows copper ion detection based on color change according to a 365 nm UV lamp (Figure 6B). In some specific scenarios, such as the detection of Zn^2+^ ions in CH_3_CN/HEPES solution (1/1, 10.0 mu M, pH = 7.0), a Schiff base fluorescent probe (L) designed by Chang *et al.* (Chang et al., 2020) can efficiently achieve this purpose. Wang *et al.* (Wang et al., 2018) devised and synthesized a novel coumarin based dual chemosensor (probe 1), which was observed by fluorescent cell imaging as a bio-imaging fluorescent sensor for detecting Zn^2+^ in human cancer cells (Figure 6C).

## 5 Probes For Sensing Toxic Metal Ions

Among all kinds of heavy metal ions, lead, cadmium and mercury ions do great harm to the environment and human beings because of their dangerous properties. Since its high toxicity directly or indirectly affect human health, it has been widely concerned in the world. These three heavy metal ions are not biodegradable and can therefore accumulate in the environment, leading to food and water contamination. Heavy metal ions and proteins (or enzymes) can have a strong interaction in the human body, so that the protein inactivity, resulting in chronic poisoning (He and Lu, 2001). People exposed to even very low amount of lead, cadmium and mercury ions can lead to diseases of various systems of the human body. Therefore, a reliable, and convenient method to detect heavy metal ions, especially Hg^2+^, Pb^2+^ and Cd^2+^, has generated a lot of interest in recent years, which is of great significance not only in environmental research, but also in food research and industry and agriculture (Kim et al., 2012).

### 5.1 Probes For Detecting Hg^2+^

The detection of mercury ions is of great importance due to their high toxicity and wide distribution in the environment, through which they can enter the food chain and then have an impact on human life and health (Staudinger and Borisov, 2015). Therefore, there is an urgent need for simple, inexpensive and reliable mercury detection methods with high selectivity and sensitivity. In recent years, fluorescence probes used to detect mercury ions are classified according to the changes of fluorescence signal, including signal attenuation fluorescence probes, signal enhancement fluorescence probes and ratio fluorescence probes (Wang Y. et al., 2021). The applications of mercury ion probes can be broadly divided into two areas: environmental and biological.

In purpose of detecting ions in the environment, Lu *et al.* (Lu et al., 2012) reported for the first time a water-soluble fluorescent carbon nanoparticles (CPs) and used to detect Hg^2+^ in natural environmental lakes, a method that is environmentally friendly, economical and simple with greater universality. In addition, in order to detect Hg^2+^ in real lake water, Zhang *et al.* (Zhang and Chen, 2014) also tried another method, where they obtained nitrogen-doped carbon quantum dots (N-CQDs) using folic acid as the carbon and nitrogen sources, which proved to be highly luminescent with a detection limit of 0.23 μM. Wang *et al.* (Wang S. et al., 2021) synthesized thioctic acid-carbon dots (SCDs), which was used as an “off-on” type fluorescent probe in the detection of Hg^2+^. Xu *et al.* (Xu et al., 2018) expected to identify Hg^2+^ by changes in fluorescence spectra and fabricated a colorimetric long-wavelength type fluorescent probe Hg-P to obtain higher selectivity. Tao *et al.* (Tao et al., 2020) synthesized a fluorescent probe based on a simple coumarin derivative, which could recognize mercury Hg^2+^ selectively in aqueous solution.

**FIGURE 7:**
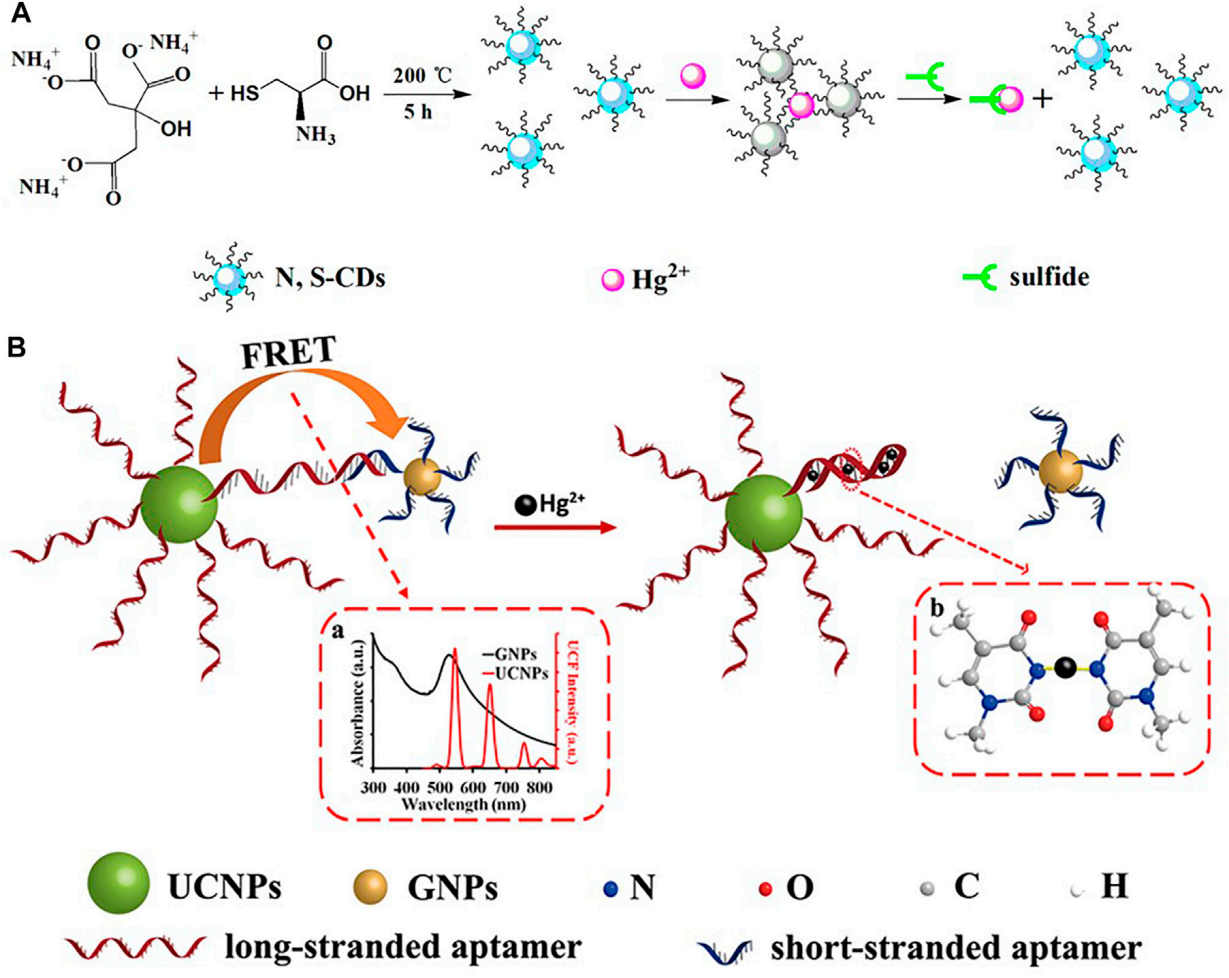
**(A)** Schematic Illustration of the Strategy for Hg^2+^ Ion and Sulfide Detection (Wu and Tong, 2019); **(B)** Schematic description of the UCNPs-aptamers-GNPs FRET sensor for Hg^2+^ (Liu et al., 2018).

In the field of biology, in order to identify Hg^2+^ in living cells, fluorescent probes with less cytotoxicity and better biocompatibility are needed, therefore Li *et al.* (Li et al., 2015) designed nitrogen-sulphur co-doped carbon dots (N,S/C-dots) and demonstrated their high fluorescence quantum yield (FLQY, 25%) and good practical application. Wu *et al.* (Wu and Tong, 2019) synthesized a nitrogen-sulfur co-doped carbon dots (N,S-CDs) as a fluorescent probe with good luminescence properties and high fluorescence quantum yield (16.1%), and achieved good results in Hg^2+^ ion recognition detection in HepG2 cells (Figure 7A). Li *et al.* (Liu et al., 2018) have done some research on nano sensors by developing a turn-on nanoprobe, which was prepared based on the principle of fluorescence resonance energy transfer (FRET) between long-chain inducer-functionalized upconversion nanoparticles (UCNPs) and short-chain inducer-functionalized gold nanoparticles (GNPs) (Figure 7B).

Pan *et al.* (Pan et al., 2018) reported the first reaction-based fluorescent probe (ATC-Hg) that detects Hg (II), which was designed based on the “covalent assembly” principle and is now used to enable the detection of Hg ions in *E. coli*. Chen *et al.* (Chen et al., 2019) synthesized a reactive fluorescent probe PIC based on peramidine group, this probe is very high in selectivity due to the fluorescence can be enhanced by 42-fold and is a promising method for the determination of mercury ions. Zhou *et al.* (Zhou et al., 2017) designed and synthesized a novel small molecule ratiometric fluorescent probe P-Hg using the ESIPT/ICT mechanism, which can be better used to identify Hg^2+^. This probe can be applied to capture mercury ions both in the natural environment and in biological systems.

Given the diversity of both environmental conditions and biological environments, the field of innovative fluorescent probes for the selective detection of Hg^2+^ faces challenges such as endogenous active substance interference, leaky cells, and photostability. Fluorescent probes are generally constructed from organic molecular framework with poor stability. Hg^2+^, as a kind of toxic heavy metal ion, seriously disrupts the natural physiological activities of living organisms. It is of great significance to design powerful probe tools to study the effects of Hg^2+^ on the health of organisms (Wang Y. et al., 2021).

### 5.2 Probes For Detecting Pb^2+^

Lead is a highly toxic substance used in batteries, gasoline and paints. Lead contamination is a chronic concern that creates a lasting threat to our human health and the environment in which we live. Even very small amounts of lead can cause serious damage to various neurological and reproductive systems in our bodies, and can even cause hypertension, lower IQ and slower reactions. According to a large study of scholars, fluorescence and colorimetric sensors were well used, and roughly divided into several categories according to its receptors, including chemical sensor based on nanoparticles, polymer, small molecules, naphthalimide and nanoparticles (Kim et al., 2012).

**FIGURE 8:**
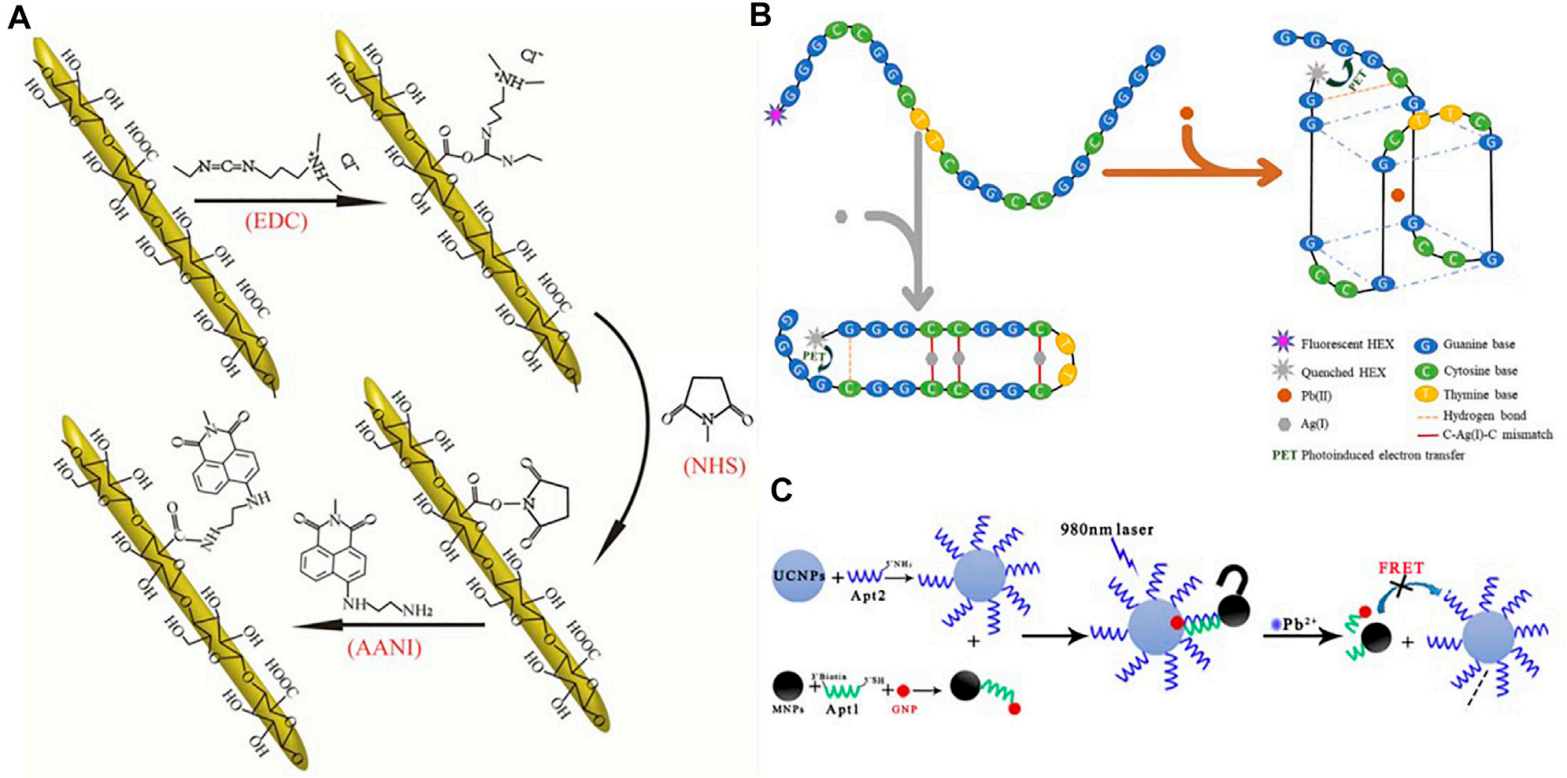
**(A)** Schematic synthesis route of the covalent immobilization of 1,8-naphthalimide dye on the surface of CNC (Song et al., 2019); **(B)** Schematic diagram for illustrating the simultaneous detection of Pb (II) and Ag (I) ions utilizing single-labelled florescent OND probe (Zhang J. et al., 2021); **(C)** a Schematic presentation of fluorescent nanoprobe based on fluorescence resonance energy transfer (FRET) between UCNPs and GNPs-MNPs for detection of Pb^2+^ (Chen et al., 2020).

A number of nanoparticle-based sensing systems have been investigated. Song *et al.* (Song et al., 2019) designed a fluorescent ion probe for achieving the measurement of lead ions in aquatic solutions using covalent binding fluorescence of 1, 8-naphthylamine dyes with cellulose nanocrystals (CNCs) (Figure 8A). Zhang *et al.* (Zhang J. et al., 2021) designed a new dual-functional oligonucleotide (OND) probe for the trace Pb (II) detection, in which the 5' end is a single fluorescent moiety using HEX labeling (Figure 8B). Chen *et al.* (Chen et al., 2020) developed a fluorometric nanoprobe for the determination of Pb (II) based on up-converted nanoparticles (UCNPs) and on magnetic Fe_3_O_4_-modified (MNPs) gold nanoparticles (GNPs) (Figure 8C).

Several sensing systems based on polymers and small molecule particles have been developed. Chini *et al.* (Chini et al., 2019) reported a polymodal sensing method consisting of a highly fluorescent dansyl-labeled copolymer P (MMA-co-Dansyl-Ala-HEMA) (DCP) and a small molecule diketopyrrolopyrrole (DPP) for the assay of the heavy metal lead (Pb^2+^). Liu *et al.* (Liu J. et al., 2013) reported a highly selective Pb^2+^ fluorescent probe that is consisted of a BODIPY fluorescent moiety and a polyamide receptor. Anand *et al.* (Anand et al., 2015) synthesized a *de novo* probe 5-[(anthracene-9-methylene) amino] quinolin-10-ol (ANQ) on the anthracene platform. What’s more, Liu *et al.*(Liu et al., 2015) reported a fluorescent turn-on probe of an oxadiazole derivative (OXD) that is based on the Schiff base molecule and was used to detect lead ions.

Fluorescent sensors based on naphthalimide are also an essential structure. Jiang *et al.* (Jiang et al., 2021) prepared a chemical fluorescent probe by attaching thiocarbamate to a naphthylamine derivative and applied it for Pb^2+^ recognition in specific chemical reagents. Un *et al.* (Un et al., 2014) successfully developed a good performance naphthylamino fluorescent probe named NPA with Pb^2+^ recognition.

Other sensors based on receptors are still available for the detection of lead ions. Recently, Bi and co-workers (Bi et al., 2017) rationally designed and developed a unique near-infrared fluorescent probe (NIR-PbP) and verified the capability of probing Pb (II) ions in both solution and live cells. Mei *et al.* (Mei et al., 2020) designed a newly available fluorescent probe L for Pb^2+^ based phenanthroline derivative. Khandare *et al.* (Khandare et al., 2014) developed a fluorescent sensor based on aggregation-induced emission (AIE) using the intense affinity of lead ions for phosphate residues. Zhao *et al.* (Zhao et al., 2016) developed a fluorescent biosensor to detect Pb^2+^ that utilized a water-soluble derivative of cationic perylene (compound 1) with the characteristics of simplicity, label-free and high speed.

### 5.3 Probes For Detecting Cd^2+^

Cd ions are also toxic to cells, while there are few fluorescent tools to study Cd^2+^ toxicity. Due to the very similar binding properties of Cd^2+^ and Zn^2+^, this poses a great challenge and difficulty for the development of Cd^2+^ probes. The first HK-2 intracellular fluorescent probe for Cd^2+^ was a Liu Cd-1 consisting of fluorescein and thiocarbamate (Carter et al., 2014). Nowadays, various methods have been well developed for the determination of Cd^2+^, such as simultaneous radiation X-ray spectrometry, synchrotron X-ray spectrometry and atomic absorption chromatography. In recent years, researches on Cd^2+^ ion fluorescent probes include the following categories: Quinolone-based, coumarin-, benzothiazole based, rhodamine-based, dansyl based, and diarylethylene based Cd^2+^ fluorescent sensor, There are also other small organic molecules based Cd^2+^ sensor and nanosensor-based Cd^2+^ fluorescent sensor. In addition, fluorescence and colorimetric nano Cd^2+^ sensors, such as metal-organic framework (MOF), quantum dots (QD), nanoclusters (NCs) and nanoparticles (NPs), have also made good progress in recent years (Shi et al., 2021).

**FIGURE 9:**
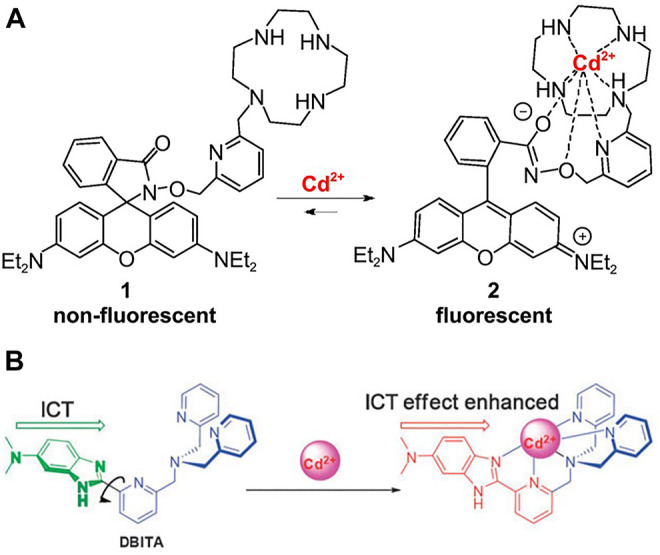
**(A)** Binding of Rhodamine-Cyclen Probe 1 with Cd^2+^ (Shim and Tae, 2011); **(B)** Proposed Cd^2+^ binding mode of DBITA (Liu et al., 2010).

First, due to the ease of synthesis and precise control of the active site (Abdollahi et al., 2020), the synthesis and design of metal-organic framework (MOF) have been applied to the design of neutral architectures for anion recognition (Esrafili et al., 2020) and the selective capture of heavy metal ions (Esrafili et al., 2021). To detect Cd^2+^ in alkaline solutions, Hao et al. (Hao and Yan, 2015) reported the one of the first fluorescent probes for Cd^2+^ on the basis of lanthanide structure of functionalized metal-organic framework (MOF), and this sensor has the advantage of a high sensitivity. Liu *et al.* (Liu Q. et al., 2013) designed a kind of new fluorescent probe DQCd_2_ for Cd^2+^ based on 4-piperidinylquinoline by utilizing the ratiometric reaction in phosphate buffered saline solution (PBS) buffer, and its emission intensity was significantly enhanced. Tsukamoto *et al.* (Tsukamoto et al., 2016) a highly practical naphthalenyl Cd^2+^ fluorescent probe, which allows a good selectivity and is suitable for a range of pH values since this method has almost no background reaction. Furthermore, Shim *et al.* (Shim and Tae, 2011) also developed a rhodamine-hydroxylamine platform-based fluorescent probe consisting of a picolinic and a cycloalkene-binding unit (Figure 9A). Xin *et al.* (Xin et al., 2013) synthesized the first fluorescent probe on the basis of difluoroborane dibenzoyl for the determination of Cd^2+^. Liu *et al.* (Liu et al., 2010) developed DBITA, a ratiofluorescent sensor that can be used for the recognition of Cd^2+^ in live cells or aqueous media, with a Cd^2+^-induced emission redshift of 53 nm (Figure 9B). Sun *et al.* (Sun et al., 2016) reported a ratio-measured and reversible fluorescent probe for the recognition of Cd^2+^ in living cells that was based on a 6-(dimethylamino) quinaldine derivative. For Cd^2+^ in living human cells, Jiang *et al.* (Jiang et al., 2014) developed a novel fluorescence probe (L) for C-3-symmetric Schiff alkaloids on the basis of 8-hydroxy-2-methylquinoline. Liu *et al.* (Liu et al., 2014) synthesized a well-performing organic salt probe in the determination of Cd^2+^ based on bis-1, 3, 4-oxadiazole derivatives and BAPTA. Liu et al. (Liu et al., 2012) developed and synthesized a novel two-photon excited cadmium fluorescent probe (named TPCd) based on o-phenylenediamine derivatives and Prodan (6-acetyl-2-methoxynaphthalene) derivatives by a two-photon approach, and demonstrated that Cd^2+^ detection in organisms is feasible and versatile. Therefore, this is a very meaningful study.

## 6 Conclusion

In summary, the identification and detection of biologically and environmentally critical species is already an important area of research in the field of the chemosensor. Fluorometry in combination with suitable probes is the preferred and excellent method of measuring these analytes due to the fast, nondestructive and sensitive nature of fluorometry measurements, and important progress has been realized in the definition and composition of fluorescent chemosensors predicated on various platforms. More and more researchers are engaged in this field, and have accumulated a lot of theoretical and practical experience in the development, synthesis and application of probes, accelerating further development of new fluorescent probes.

In this paper, the research progress of fluorescent probes for metal ion detection is reviewed. These fluorescent probes are mainly divided into organic small molecule probes and nano fluorescent probes. For different metal ions, different materials are selected to design and synthesize fluorescent probes, such as quantum dots, coumarin derivatives, benzene derivatives, etc. The development of sensing mechanisms plays an important role in improving the sensing performance of metal ions. For example, the solar excited naked eye Fe^3+^ detection is realized by improving the luminescence efficiency based on the central hydrophobic/external hydrophilic strategy (Sun et al., 2020). So far, the main efforts have been devoted to the study of new sensing mechanisms, strategies to expand the range of detected metal ions, and methods to improve sensitivity and selectivity. However, there are still limitations and unparalleled challenges in the practical application of fluorescent probes. The variation of pH, temperature and probe behavior in different environments, as well as the accompanying fluorescence burst effect in some fluorescent sensors, have raised high requirements for the design of fluorescent probes. Therefore, in future, lots of aspects need to be further improved, including compatibility of probes in organisms and accuracy of fluorescent probe sensing, etc., so they can be applicable to more complex detection environments. We believe that fluorescent probes will become a very powerful tool in the biomedical field and make great contributions to biology in future.
